# Resveratrol Mitigates Bisphenol A-Induced Metabolic Disruptions: Insights from Experimental Studies

**DOI:** 10.3390/molecules28155865

**Published:** 2023-08-03

**Authors:** Muhammad Sajid Hamid Akash, Mutayyba Fatima, Kanwal Rehman, Qudsia Rehman, Zunera Chauhdary, Ahmed Nadeem, Tahir Maqbool Mir

**Affiliations:** 1 Department of Pharmaceutical Chemistry, Government College University, Faisalabad 38000, Pakistan; 2 Department of Pharmacy, The Women University, Multan 60000, Pakistan; 3 Department of Pharmacology and Toxicology, College of Pharmacy, King Saud University, Riyadh 11451, Saudi Arabia; 4 National Center for Natural Products Research, School of Pharmacy, University of Mississippi, University, MS 38677, USA

**Keywords:** insulin growth factor 1, Glucokinase, metabolic disorders, endocrine disruptor, uncoupling protein 2, RT-qPCR

## Abstract

The aim of this study was to investigate the disruptions of metabolic pathways induced by bisphenol A (BPA) and explore the potential therapeutic intervention provided by resveratrol (RSV) in mitigating these disruptions through the modulation of biochemical pathways. Wistar albino rats were divided into three groups: group 1 served as the control, group 2 received 70 mg/Kg of BPA, and group 3 received 70 mg/kg of BPA along with 100 mg/Kg of RSV. After the treatment period, various biomarkers and gene expressions were measured to assess the effects of BPA and the potential protective effects of RSV. The results revealed that BPA exposure significantly increased the serum levels of α-amylase, α-glucosidase, G6PC, insulin, HbA1c, HMG-CoA reductase, FFAs, TGs, DPP-4, MDA, and proinflammatory cytokines such as TNF-α and IL-6. Concurrently, BPA exposure led to a reduction in the levels of antioxidant enzymes such as catalase (CAT), glutathione peroxidase (GPx), and superoxide dismutase (SOD), as well as GLUT4 and HDL cholesterol. However, the administration of RSV along with BPA significantly ameliorated these alterations in the biomarker levels induced through BPA exposure. RSV treatment effectively reduced the elevated levels of α-amylase, α-glucosidase, G6PC, insulin, HbA1c, HMG-CoA reductase, FFAs, TGs, DPP-4, MDA, and proinflammatory cytokines, while increasing the levels of antioxidant enzymes, GLUT4, and HDL cholesterol. Furthermore, BPA exposure suppressed the mRNA expression of glucokinase (GCK), insulin-like growth factor 1 (IGF-1), and glucose transporter 2 (GLUT2) and up-regulated the mRNA expression of uncoupling protein 2 (UCP2), which are all critical biomarkers involved in glucose metabolism and insulin regulation. In contrast, RSV treatment effectively restored the altered mRNA expressions of these biomarkers, indicating its potential to modulate transcriptional pathways and restore normal metabolic function. In conclusion, the findings of this study strongly suggest that RSV holds promise as a therapeutic intervention for BPA-induced metabolic disorders. By mitigating the disruptions in various metabolic pathways and modulating gene expressions related to glucose metabolism and insulin regulation, RSV shows potential in restoring normal metabolic function and counteracting the adverse effects induced by BPA exposure. However, further research is necessary to fully understand the underlying mechanisms and optimize the dosage and duration of RSV treatment for maximum therapeutic benefits.

## 1. Introduction

Bisphenol A (BPA) is an extensively used chemical compound, primarily serving as a monomer in the manufacturing of polycarbonate plastics and epoxy resins [1]. These materials are commonly employed in the production of various everyday items such as food containers, water bottles, milk containers, tin can liners, and numerous household products [2]. BPA is also present in dental sealants, further expanding its range of applications. This compound, while economically significant, has gained attention as an endocrine-disrupting chemical (EDC) due to its adverse effects on metabolic functions within the human body [3]. Numerous studies have established a link between BPA exposure and the impairment of several metabolic processes, including the development of conditions such as diabetes mellitus (DM), obesity, and metabolic syndromes [3,4,5,6,7,8]. The disruption of metabolic pathways with BPA can have significant implications for human health [2]. Studies have indicated that even at low doses, BPA can interfere with carbohydrate metabolism, impair insulin secretion and sensitivity, and contribute to the development of insulin resistance [9]. Additionally, BPA-induced oxidative stress has been linked to the dysregulation of lipid metabolism, leading to abnormal lipid profiles and increased risk of cardiovascular diseases [1,10]. The precise mechanisms through which this occurs have been the subject of scientific inquiry. Additionally, experimental studies have focused on the impact of BPA on the functionality of β-cells and α-cells found in the islets of Langerhans, which are responsible for the secretion of insulin and glucagon, respectively [11]. These investigations have provided evidence that BPA can interfere with the normal functioning of these cells, potentially leading to imbalances in the regulation of glucose levels and hormone secretion [2]. Overall, the utilization of BPA in various consumer products raises concerns regarding its potential detrimental effects on metabolic health and endocrine function [12]. Extensive research efforts continue to delve into the mechanisms and consequences of BPA exposure in order to better understand and address the associated risks to human health. Accumulating evidence suggests that BPA exposure is associated with the development of metabolic disorders, including DM, obesity, and metabolic syndromes [13].

The detrimental effects of BPA on metabolic functions have been extensively investigated in both in vitro and in vivo studies [14]. Experimental evidence has revealed that BPA can disrupt the functionality of pancreatic β-cells and α-cells, which are responsible for insulin and glucagon secretion, respectively [11]. The dysregulation of these cells can lead to impaired glucose metabolism and insulin resistance, contributing to the development of DM and related metabolic disorders [15]. Furthermore, BPA-induced oxidative stress plays a critical role in the pathogenesis of metabolic disorders [16]. Lipid peroxidation, resulting from the oxidation of lipoproteins and lipids, can overwhelm the antioxidant defense system, induce cellular apoptosis, and elevate the production of ROS [17]. Elevated ROS levels can further exacerbate insulin resistance, impair pancreatic β-cell function, and promote inflammation and tissue damage [18]. Given the alarming rise in BPA exposure and its potential health risks, there is a need to explore therapeutic interventions that can counteract the disruptions caused by BPA on metabolic pathways [19].

Maintaining a delicate balance between the oxidation and reduction of chemicals is crucial for various aspects of cellular development, growth, and survival [20]. Reactive oxygen species (ROS), including peroxides, O^2−^, and OH radicals, are naturally produced as byproducts of cellular metabolism [21,22]. Cells possess tightly regulated pathways to utilize small amounts of these radicals for gene regulation while effectively clearing excessive ROS to prevent detrimental effects [22]. Disruptions in this balance can render cells insensitive to cell death signals triggered by environmental toxins, leading to genetic mutations and alterations in gene expression [23,24]. Thus, the intricate management of ROS levels is essential for maintaining cellular health and preventing oxidative-stress-related damage [25].

Resveratrol (RSV), a member of the polyphenol class, possesses antioxidant properties and plays a role in reducing cellular oxidative stress [26]. RSV has emerged as a potential candidate due to its ability to modulate gene expressions related to glucose metabolism, enhance insulin sensitivity, and mitigate oxidative stress [26]. This compound can be found naturally in various sources such as blueberries, mulberries, raspberries, and peanuts, and is particularly abundant in the peel and seeds of red grapes [27]. The beneficial properties of RSV are wide-ranging and include antioxidant, antidiabetic, antiadipogenic, anti-inflammatory, antitumor, and antimicrobial activities [28]. RSV exists in two interconvertible isomeric forms [29], with trans-resveratrol exhibiting greater stability compared to cis-resveratrol [30,31,32]. Previous research has demonstrated that RSV can improve glucose homeostasis, enhance insulin sensitivity, and ameliorate oxidative stress in various experimental models [33]. These beneficial effects of RSV make it a promising candidate for mitigating the adverse effects induced by BPA exposure.

In this study, we aimed to investigate the disruptions of metabolic pathways induced by BPA and explore the potential therapeutic intervention provided by RSV. We assessed the effects of BPA exposure on various biomarkers related to glucose metabolism, lipid metabolism, oxidative stress, and inflammation. Additionally, we examined the gene expressions of key biomarkers involved in glucose metabolism and insulin regulation. By examining the effects of resveratrol on BPA-induced metabolic dysfunction, we aimed to uncover its ability to counteract the detrimental effects of BPA and potentially provide a therapeutic intervention for individuals exposed to BPA-related metabolic disturbances.

## 2. Results

### 2.1. Analysis of Anthropometric Biomarkers

**Figure 1:**
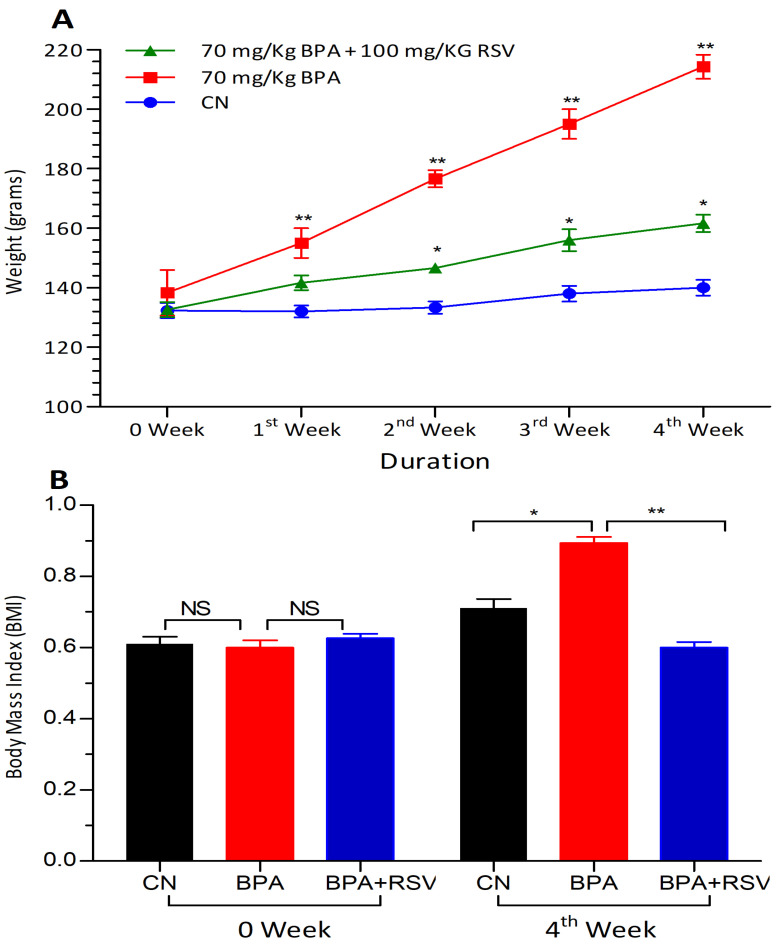
Effect of treatment on (**A**) weight and (**B**) BMI. Data analyzed with two-way ANOVA, and Bonferroni’s multiple comparison test was used as a posttest to compare all pairs of columns; * indicates *p* < 0.05 when compared with control group, ** indicates *p* < 0.05 when compared with BPA treated group with control and RSV treated group, respectively.

In this study, the weight and BMI of the rats were measured to explore the potential effects of BPA on obesity. The weight of the rats served as an indicator of overall body mass, while the BMI provided a measure of body weight adjusted for the size of the rats. The results showed that the groups exposed to BPA alone exhibited a significant increase (*p* < 0.05) in both body weight and BMI values compared to the control group. This finding suggests that BPA exposure may contribute to weight gain and an increase in BMI, potentially indicating a role in the development of obesity. However, when comparing the total body weight values of rats treated with both RSV and BPA to the control group, no significant difference was observed. This suggests that the administration of RSV alongside BPA may have mitigated the weight gain typically associated with BPA exposure. The lack of a significant difference between the RSV+BPA group and the control group in terms of body weight implies a potential protective effect of RSV against the adverse effects of BPA on weight gain. These findings are visually represented in Figure 1, which illustrates the comparison of the different groups’ total body weight values. By measuring weight and BMI, this study provides valuable insights into the potential side effects of BPA on obesity development and highlights the potential protective role of RSV in mitigating these effects.

### 2.2. Analysis of Glycemic Control Biomarkers

**Figure 2:**
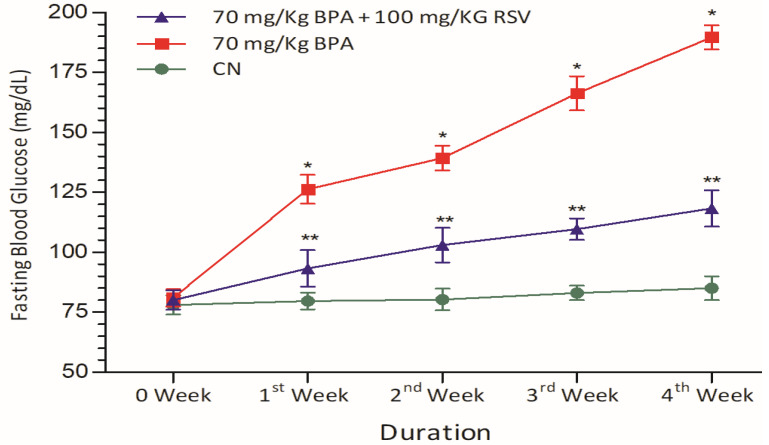
Effect of treatment on fasting blood glucose levels. Data analyzed with two-way ANOVA, and Bonferroni’s multiple comparison test was used as a posttest to compare all pairs of columns; * indicates *p* < 0.05 when compared with control group, ** indicates *p* < 0.05 when compared with BPA-exposed group.

The primary objective of this study was to investigate the diabetogenic effect of BPA in Wistar albino rats by examining glycemic control biomarkers. The rats exposed to BPA exhibited a significant increase in blood glucose levels compared to the control group, as depicted in Figure 2. This finding suggests that BPA exposure can disrupt glycemic control and lead to elevated blood glucose levels, which are characteristic of diabetes. However, the co-administration of RSV significantly mitigated the elevated blood glucose levels when compared to rats exposed solely to BPA. This indicates that RSV may have a protective effect against the diabetogenic effects of BPA, as shown in Figure 2. In addition to blood glucose levels, the study also assessed the serum levels of HbA1c and DPP-4, which are additional indicators of glycemic control and DM. The findings demonstrated that BPA exposure led to an increase in the serum levels of HbA1c, as illustrated in Figure 2. This elevation in HbA1c levels indicates poor glycemic control and is a hallmark of DM.

**Figure 3:**
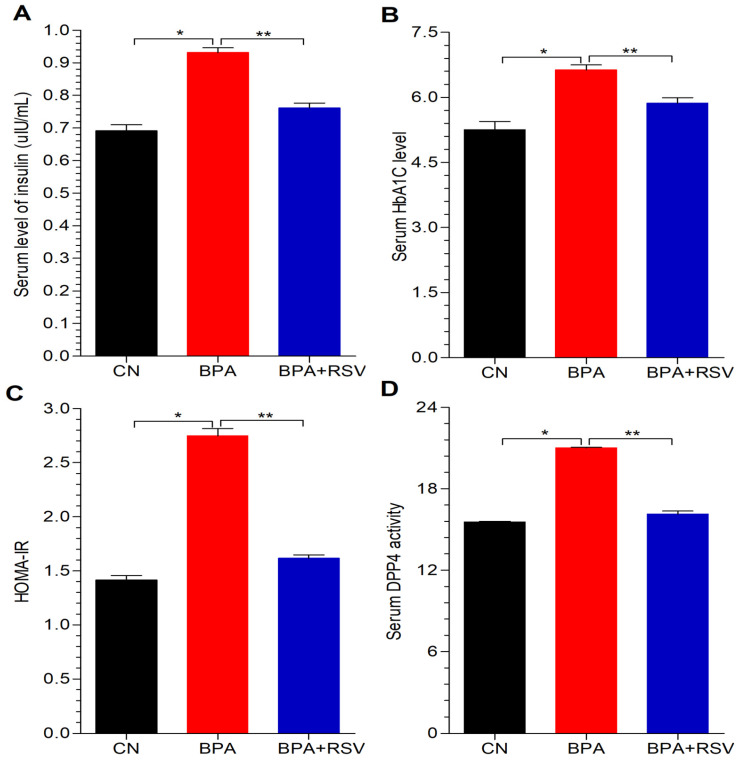
Effect of treatment on (**A**) insulin, (**B**) HbA1c, (**C**) HOMA-IR, and (**D**) DPP-4. Data analyzed with one-way ANOVA, and Bonferroni’s multiple comparison test was used as a posttest to compare all pairs of columns; * indicates *p* < 0.05 when compared with control group, ** indicates *p* < 0.05 when compared with BPA-exposed group.

The increased levels of DPP-4 (Figure 3D) further support the adverse effects of BPA on glycemic control, as DPP-4 is involved in glucose metabolism and its elevated levels are associated with impaired glucose regulation. Moreover, the serum level of insulin was found to be significantly higher in the experimental rats exposed to BPA (Figure 3A). This observation suggests that BPA exposure may lead to insulin resistance impairing glucose uptake and utilization. This is further supported by the increased value of HOMA-IR (Figure 3C), which is a marker of insulin resistance. Taken together, these results indicate that BPA exposure has adverse effects on various glycemic control biomarkers, including blood glucose levels, HbA1c, DPP-4, insulin, and HOMA-IR. These biomarkers collectively indicate the diabetogenic potential of BPA in Wistar albino rats, suggesting that BPA exposure may contribute to the development or worsening of diabetes-like conditions in this animal model.

### 2.3. Analysis of Lipid Profile Biomarkers

**Figure 4:**
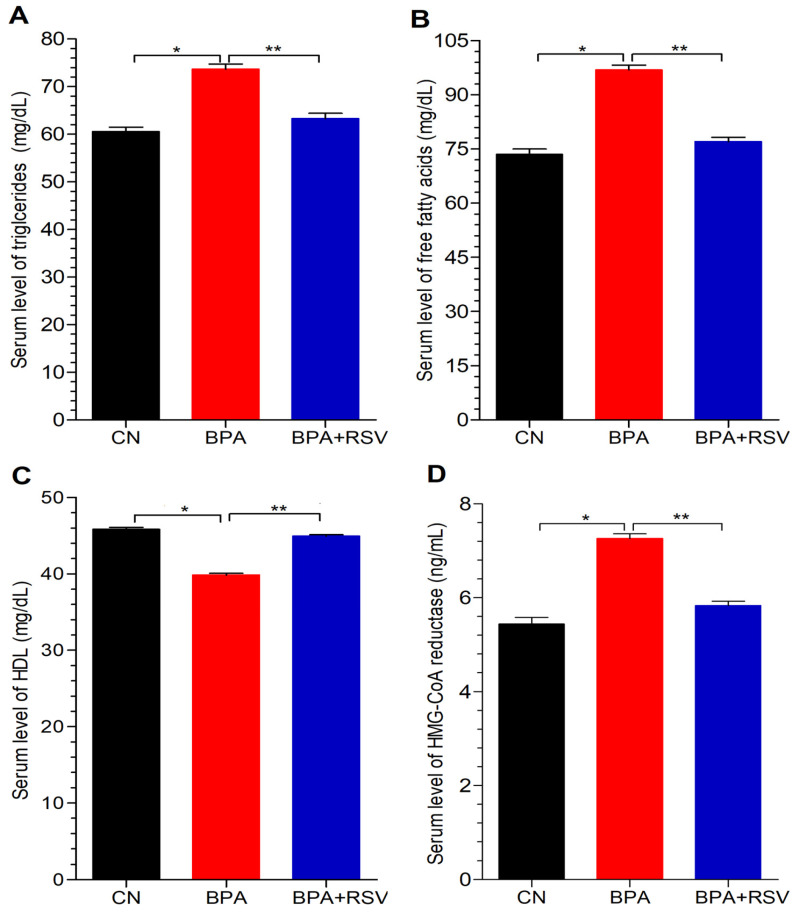
Effect of treatment on (**A**) TGs, (**B**) FFAs, (**C**) HDL, and (**D**) HMG-CoA reductase. Data analyzed with one-way ANOVA, and Bonferroni’s multiple comparison test was used as a posttest to compare all pairs of columns; * indicates *p* < 0.05 when compared with control group, ** indicates *p* < 0.05 when compared with BPA-exposed group.

To investigate the impact of BPA on lipid biomarkers, this study focused on measuring serum levels of TGs, FFAs, and HDL. The results revealed that rats exposed to BPA exhibited a significant increase (*p* < 0.05) in both TGs (Figure 4A) and FFAs (Figure 4B) compared to the control group. However, the co-administration of RSV effectively mitigated the elevated levels of both TGs and FFAs when compared to rats exposed solely to BPA. This indicates that RSV has a beneficial effect in reducing the heightened levels of TGs and FFAs induced by BPA, as depicted in Figure 4A and B. Conversely, BPA exposure had a negative effect on the serum level of HDL. The group exposed to BPA exhibited noticeably higher levels of HDL compared to the control group (Figure 4C). However, the administration of RSV resulted in an improvement in the serum levels of HDL, indicating a positive effect on HDL levels (Figure 4C). These findings highlight the significant impacts of BPA exposure on lipid biomarkers. The elevated levels of TGs and FFAs and the decrease in HDL levels observed in the BPA-exposed group indicate an unfavorable lipid profile that may contribute to the development of dyslipidemia and associated cardiovascular risks. However, the concurrent administration of RSV demonstrated a beneficial effect by reducing the heightened levels of TGs and FFAs, as well as improving HDL levels. This suggests that RSV supplementation has the potential to mitigate the negative effects of BPA on lipid metabolism and promote a healthier lipid profile.

### 2.4. Analysis of Cholesterol Biosynthesis

HMG-CoA reductase is a key enzyme involved in the mevalonate pathway, which is responsible for cholesterol biosynthesis in the body. This enzyme serves as a rate-limiting step in the production of cholesterol. The results indicated that rats exposed to BPA exhibited a significant increase (*p* < 0.05) in the level of HMG-CoA reductase compared to both the control group and the RSV-treated group, as shown in Figure 4D. This finding suggests that BPA exposure has a stimulatory effect on the expression or activity of HMG-CoA reductase in the liver. However, no significant difference in HMG-CoA reductase levels was observed between the control group and the RSV-treated group. This suggests that RSV treatment did not influence the levels of HMG-CoA reductase in the liver (Figure 4D). The absence of a significant difference between the control group and the RSV-treated group indicates that RSV did not modulate the activity or expression of HMG-CoA reductase. These results provide valuable insights into the potential mechanism through which BPA affects cholesterol biosynthesis through the modulation of HMG-CoA reductase activity. The significant increase in HMG-CoA reductase levels in hepatic tissues of BPA-exposed rats suggests that BPA may enhance the production of cholesterol by upregulating the activity or expression of this key enzyme.

### 2.5. Analysis of Carbohydrate Metabolism

**Figure 5:**
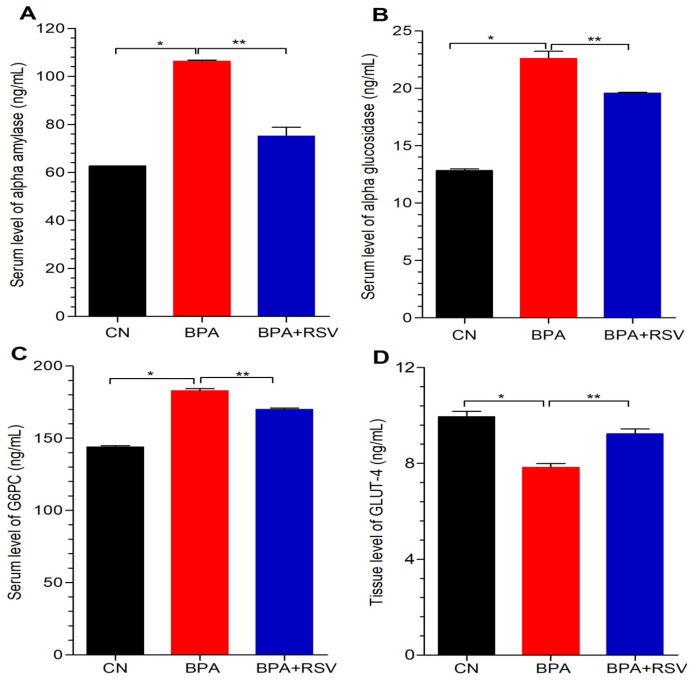
Effect of treatment on (**A**) alpha amylase, (**B**) alpha glucosidase, (**C**) G6PC = glucose 6 Phosphatase, and (**D**) GLUT4 = glucose transporters 4. Data analyzed with one-way ANOVA, and Bonferroni’s multiple comparison test was used as a posttest to compare all pairs of columns; * indicates *p* < 0.05 when compared with control group, ** indicates *p* < 0.05 when compared with BPA-exposed group.

In our study, we aimed to investigate the impact of BPA exposure on carbohydrate metabolism by analyzing the serum levels of key enzymes involved in this process. Specifically, we focused on α-amylase, α-glucosidase, and glucose-6-phosphatase, which are critical enzymes in carbohydrate digestion, breakdown, and glucose production, respectively. The results demonstrated a significant elevation (*p* < 0.05) in the serum levels of α-amylase (Figure 5A), α-glucosidase (Figure 5B), and glucose-6-phosphatase (Figure 5C) in the BPA-exposed group compared to the control group. This increase in enzyme levels indicates a disruption in normal carbohydrate metabolism caused by BPA exposure. Elevated levels of these enzymes are known to contribute to hyperglycemic effects, suggesting that BPA may induce an imbalance in glucose homeostasis and impair carbohydrate metabolism. In contrast, when the BPA-exposed group was treated with RSV, there was a significant decrease (*p* < 0.05) in the serum levels of α-amylase, α-glucosidase, and glucose-6-phosphatase. This finding suggests that RSV effectively prevented the impaired carbohydrate metabolism induced by BPA. The reduction in enzyme levels in the BPA and RSV co-treatment group indicates the potential of RSV to counteract the detrimental effects of BPA on carbohydrate metabolism. These findings provide substantial evidence confirming the detrimental impact of BPA exposure on carbohydrate metabolism, as evidenced by the elevated levels of α-amylase, α-glucosidase, and glucose-6-phosphatase. The significant reduction in enzyme levels observed in the BPA and RSV co-treatment group further emphasizes the preventive effect of RSV on BPA-induced disruptions in carbohydrate metabolism.

### 2.6. Analysis of Insulin-Regulated Glucose Transporter

In addition to assessing carbohydrate metabolism enzymes, we also investigated the levels of GLUT4 in skeletal muscle tissues to gain further insights into the impact of BPA exposure on insulin signaling and glucose uptake. GLUT4 is a key protein involved in insulin-mediated glucose transport in skeletal muscle cells. Our analysis revealed a significant decrease (*p* < 0.05) in GLUT4 levels in the BPA-exposed group compared to the control group, as depicted in Figure 5D. This finding indicates that BPA exposure impairs insulin signaling through the Akt/GLUT4 pathways, leading to a reduction in glucose uptake in skeletal muscle tissues. The decreased levels of GLUT4 suggest a disruption in the normal insulin-mediated glucose transport process, potentially contributing to insulin resistance and impaired glucose homeostasis. Interestingly, there was no significant difference between the control group and the group receiving co-administration of RSV with BPA. This suggests that RSV effectively prevents the development of insulin resistance associated with BPA exposure, as it did not impact GLUT4 levels. The maintenance of GLUT4 levels in the RSV and BPA co-administration group implies that RSV may counteract the negative effects of BPA on insulin signaling and glucose uptake in skeletal muscle tissues. These results provide important insights into the underlying mechanism by which BPA affects insulin signaling and glucose uptake through the regulation of GLUT4 levels in skeletal muscle tissues. The significant decrease in GLUT4 levels observed in the BPA-exposed group supports the notion of disrupted insulin-mediated glucose transport.

### 2.7. Analysis of Biomarkers of Oxidative Stress

**Figure 6:**
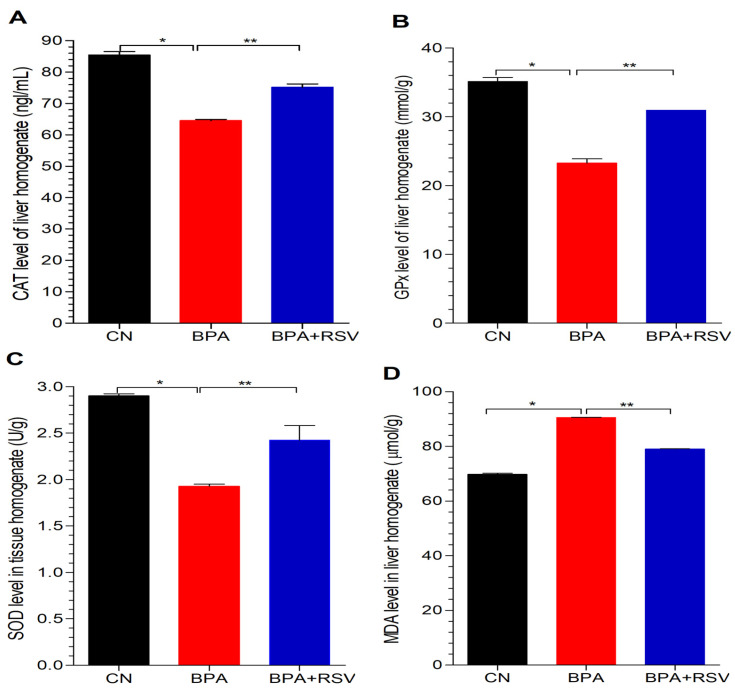
Effect of treatment on (**A**) CAT = catalase, (**B**) GSH = glutathione, (**C**) SOD = superoxide dismutase, and (**D**) MDA = Malondialdehyde. Data analyzed with one-way ANOVA, and Bonferroni’s multiple comparison test was used as a posttest to compare all pairs of columns; * indicates *p* < 0.05 when compared with control group, ** indicates *p* < 0.05 when compared with BPA-exposed group.

In our study, we investigated the effects of BPA exposure on oxidative stress and lipid peroxidation in liver tissue homogenates. Oxidative stress occurs when there is an imbalance between the production of ROS and the antioxidant defense system in the body. We observed that BPA exposure resulted in a significant reduction (*p* < 0.05) in the levels of antioxidants, including CAT, GPx, and SOD, in liver tissue homogenates compared to the control group (Figure 6). This reduction in antioxidant levels indicates an impaired antioxidant defense system, leading to increased oxidative stress in the liver. Conversely, BPA exposure significantly increased (*p* < 0.05) the levels of MDA (Figure 6D). However, when RSV was co-administered at a dose of 100 mg, it exhibited a protective effect against the imbalanced oxidative status induced by BPA. In the BPA and RSV co-administration group, significant increases (*p* < 0.05) were observed in CAT and GPx levels, as well as a significant increase (*p* < 0.05) in SOD levels in liver tissue homogenates compared to the BPA-treated group. These results suggest that RSV supplementation can restore the levels of antioxidants and enhance the antioxidant defense system in liver tissues. Furthermore, RSV demonstrated the ability to reduce lipid peroxidation, as it significantly decreased (*p* < 0.05) MDA levels in the RSV-treated group compared to the group treated with BPA alone (Figure 6D). This finding indicates that RSV has the potential to attenuate lipid peroxidation and protect against oxidative damage to lipids in the liver.

### 2.8. Analysis of Inflammatory Biomarkers

**Figure 7:**
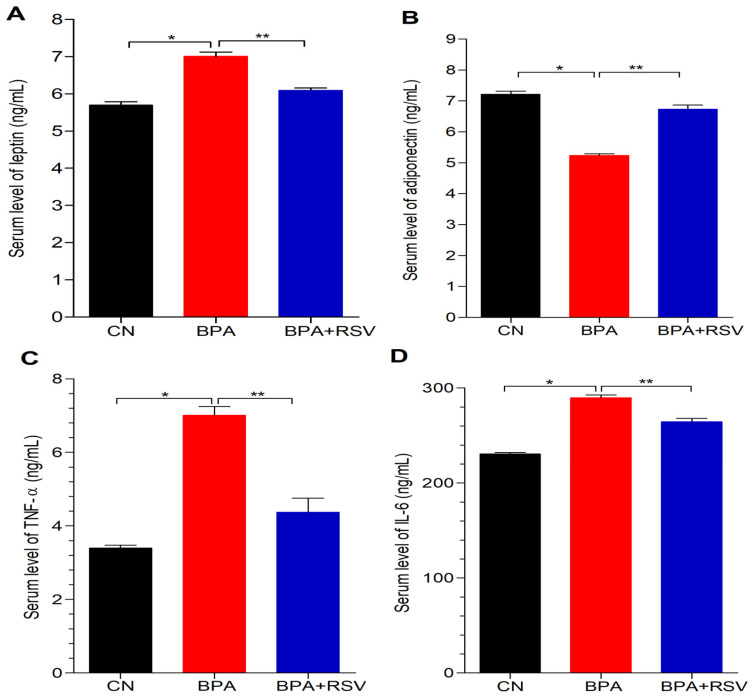
Effect of treatment on (**A**) leptin, (**B**) adiponectin, (**C**) TNF-α, and (**D**) IL-6. Data analyzed with one-way ANOVA, and Bonferroni’s multiple comparison test was used as a posttest to compare all pairs of columns; * indicates *p* < 0.05 when compared with control group, ** indicates *p* < 0.05 when compared with BPA-exposed group.

We assessed the levels of inflammatory adipocytokines, including leptin, adiponectin, TNF-α, and IL-6, in the serum. As depicted in Figure 7, the group treated with BPA alone exhibited a significant increase (*p* < 0.05) in the serum levels of leptin (Figure 7A), TNF-α (Figure 7C), and IL-6 (Figure 7D), indicating an inflammatory response compared to the control group. On the other hand, the level of adiponectin (Figure 7B), which is known for its anti-inflammatory properties, was significantly decreased (*p* < 0.05) in the BPA-treated group compared to the control group. These findings suggest that BPA exposure induces an inflammatory state characterized by elevated levels of pro-inflammatory adipocytokines and reduced levels of anti-inflammatory adiponectin. Remarkably, RSV treatment demonstrated the potential to mitigate the elevation of serum levels of inflammatory adipocytokines induced by BPA. In the RSV-BPA treated group, RSV significantly decreased (*p* < 0.05) the levels of leptin, TNF-α, and IL-6 in the serum, while significantly increasing (*p* < 0.05) the levels of adiponectin compared to the BPA-treated group. These results indicate that RSV has the ability to restore the balance of adipocytokines and alleviate the BPA-induced inflammatory response. The significant reduction in the levels of leptin, TNF-α, and IL-6, along with the increase in adiponectin levels in the RSV-BPA treated group, show the anti-inflammatory effects of RSV against BPA-induced toxicity. The significant reduction in pro-inflammatory adipocytokines and the increase in anti-inflammatory adiponectin levels in the RSV-BPA treated group show the anti-inflammatory effects of RSV against BPA-induced toxicity.

### 2.9. Analysis of the Transcriptional Regulation of Glucose Metabolism and Insulin Stimulation

**Figure 8:**
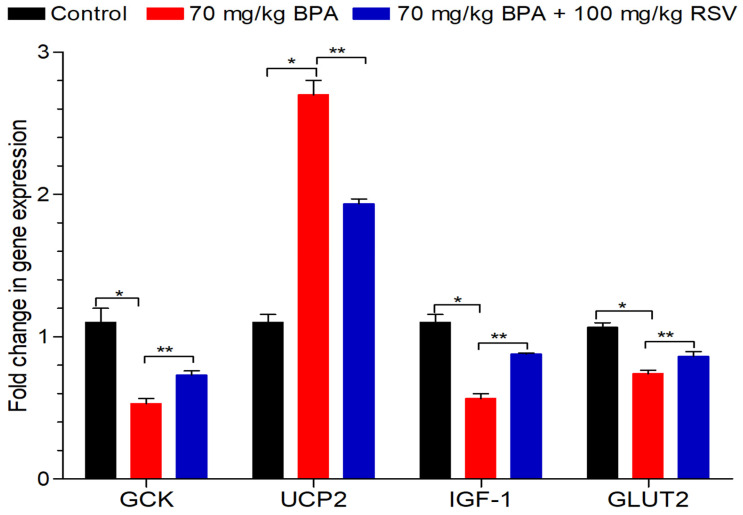
Effect of BPA and RSV on mRNA expression (GCK, UCP2, IGF-1, and GLUT2). Data analyzed with two-way ANOVA, and Bonferroni’s multiple comparison test was used as a posttest to compare all pairs of columns; * indicates *p* < 0.05 when compared with control group, ** indicates *p* < 0.05 when compared with BPA-exposed group.

To gain a clearer understanding of the disruptions in carbohydrate metabolism induced by BPA exposure, we investigated the gene expressions involved in this process using RT-qPCR. We analyzed the mRNA expression levels of several key genes, including GCK, IGF-1, GLUT2, and UCP2. As depicted in Figure 8, the group treated with BPA exhibited significant down-regulation (*p* < 0.05) of GCK, IGF-1, and GLUT2 mRNA expression levels compared to the control group. The fold changes were 0.53, 0.57, and 0.74, respectively, indicating a decrease in the expression of these genes involved in carbohydrate metabolism. Conversely, the mRNA expression level of UCP2 was significantly up-regulated (*p* < 0.05) in the BPA-treated group, with a fold change of 2.46 compared to the control group. UCP2 is known to be involved in the regulation of energy metabolism and has been associated with disruptions in carbohydrate metabolism. Remarkably, the co-administration of RSV with BPA resulted in a significant increase (*p* < 0.05) in the expression of GCK and a significant decrease in the expression of UCP2 compared to the BPA-treated group. The fold changes were 0.73 and 1.94, respectively. Moreover, RSV administration also caused a significant increase (*p* < 0.05) in the expression of IGF-1 and GLUT2 compared to the BPA-treated group, with fold changes of 0.88 and 0.83, respectively. These findings highlight that treatment with RSV mitigates the detrimental effects of BPA in rats, as evidenced by the modulation of gene expressions involved in carbohydrate metabolism. The up-regulation of GCK, IGF-1, and GLUT2, along with the downregulation of UCP2, show the beneficial effects of RSV in restoring the disrupted carbohydrate metabolism induced through BPA treatment.

### 2.10. Histopathological Analysis

**Figure 9:**
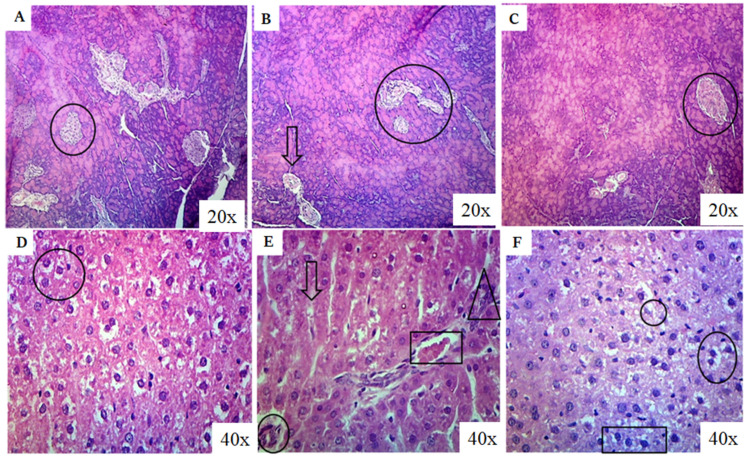
Photomicrographs of pancreas (**A**–**C**) and liver (**D**–**F**). (**A**) The circle is showing normal pancreatic appearance as Islets of Langerhans are present throughout the parenchyma which is an indication of active pancreatic parenchyma. (**B**) The arrow indicates that Islets of Langerhans are less in number which indicates necrotic changes in the pancreas. (**C**) The circle indicates the normal appearance of pancreas. (**D**) The circle shows the normal appearance of hepatic parenchyma and nuclei of the hepatocytes. (**E**) The arrow indicates vacuolar degradation and hazy vacuole, the circle is indicating perivascular cuffing, the triangle is indicating inflammatory zone, and the rectangle is indicating periportal fibrosis. (**F**) The circle indicates the normal appearance of nuclei and rectangle is showing condensed and pyknotic nuclei.

The photomicrographs of the pancreas in the control group revealed a normal appearance, with Islets of Langerhans distributed throughout the parenchyma, indicating an active pancreatic function (Figure 9A). The Islets of Langerhans are responsible for producing and secreting hormones, including insulin, essential for glucose regulation. However, in the BPA-exposed group, the Islets of Langerhans were noticeably reduced in number, suggesting necrotic changes in the pancreas (Figure 9B). This reduction in the Islets of Langerhans indicates impaired pancreatic function and a potential disruption in insulin production. In contrast, the group treated with both BPA and RSV exhibited predominantly active and enlarged Islets of Langerhans (Figure 9C), indicating a restorative effect of RSV on pancreatic tissue. This restoration of the Islets of Langerhans suggests that RSV administration may help protect and restore pancreatic function in the presence of BPA-induced toxicity. Moving on to the hepatic parenchyma (liver tissue), the control group exhibited a normal appearance (Figure 9D). Hepatocytes, the main functional cells of the liver, were organized in hepatic cords, and their nuclei appeared normal, displaying nucleoli and chromatin material. This normal appearance indicates healthy liver tissue. In the BPA-treated group, mild to moderate necrotic changes were observed in the hepatic parenchyma (Figure 9E). The nuclei of hepatocytes showed condensation and pyknosis, indicating necrotic changes. Additional pathological features included mild congestion, fibrotic changes, inflammatory zones, perivascular cuffing, and periportal fibrosis. Furthermore, mild to moderate vacuolar degradation with hazy vacuoles, individual cell necrosis, and mild inflammatory infiltrates were also observed. These histopathological changes in the liver indicate BPA-induced liver damage, including necrosis, inflammation, and fibrotic changes. In the group treated with both RSV and BPA, the hepatic parenchyma showed a moderate degree of amelioration (Figure 9F). Although the nuclei of hepatocytes were normal in some areas, condensed and pyknotic nuclei were still observed in other areas, indicating partial amelioration. This suggests that RSV administration has a protective effect on the liver tissue, partially mitigating the necrotic changes induced by BPA exposure. These observations provide a comprehensive view of the histopathological changes in the pancreas and liver caused by BPA exposure and the potential protective effects of RSV. The restoration of Islets of Langerhans in the pancreas and partial improvement in hepatic tissue suggest the ameliorative properties of RSV in mitigating the adverse effects induced by BPA. The findings indicate that RSV may help preserve pancreatic function and protect against BPA-induced liver damage, although further investigations are necessary to fully understand the mechanisms involved.

**Figure 10:**
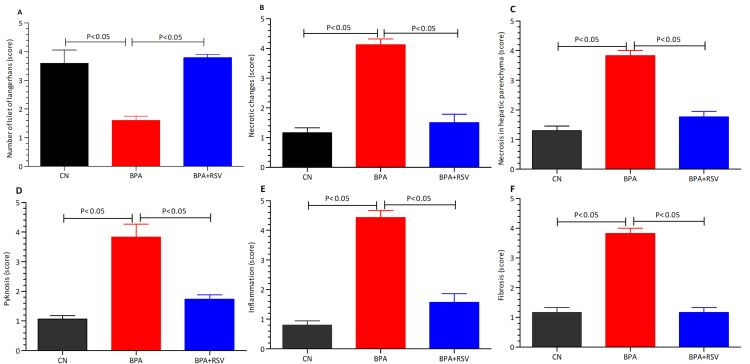
Quantitative analysis of histopathological examination. (**A**) Number of islets of Langerhans, (**B**), Necrotic changes, (**C**) necrosis in hepatic parenchyma, (**D**) pyknosis, (**E**) inflammation and (**F**) fibrosis. Data analyzed with one-way ANOVA, and Bonferroni’s multiple comparison test was used as a posttest to compare all pairs of columns.

The quantitative results of the histopathological examination are presented in Figure 10. These findings reveal that the number and size of active Islet of Langerhans score significantly decreased (*p* < 0.05) in the BPA-exposed group compared to both the control group and the RSV + BPA treatment group. Furthermore, the scores for necrotic changes, inflammatory cell infiltration, fibrosis, and pyknosis hallmarks were significantly higher (*p* < 0.05) in the BPA exposure group compared to both the control group and the RSV + BPA treatment group. These results indicate that BPA exposure leads to notable adverse effects on the Islet of Langerhans, including reduced activity and size, as well as increased necrotic changes, inflammation, fibrosis, and pyknosis. In contrast, treatment with RSV in combination with BPA appears to offer some protection against these pathological changes, as evident by the lower scores observed in the RSV + BPA treatment group. These quantitative results provide a comprehensive understanding of the histopathological alterations induced by BPA and the potential beneficial effects of RSV in mitigating its adverse impacts on the Islet of Langerhans. These findings further underscore the significance of our study and its potential implications in the field of research related to BPA toxicity and its management with RSV intervention.

## 3. Materials and Methods

### 3.1. Chemicals and Assay Kits

Insulin ELISA kit (catalogue number: IS130D, Calbiotech, Inc., El Cajon, CA, USA), HbA1c ELISA kit (catalogue number: SG 10984, Elabsciences, Wuhan, China), non-esterified free fatty acid ELISA kit (catalogue number: E-BC-K013, Elabsciences), HDL ELISA kit (catalogue number: E-BC-K222-S, Elabsciences), cholesterol ELISA kit (catalogue number: BD090618, Human diagnostics, Ahrensburg, German), triglyceride ELISA kit (catalogue number: BD090618, bioactive, Bangkok, Thailand.), HMG-CoA reductase ELISA kit (catalogue number: E-EL-H2472 Elabsciences), leptin ELISA kit (catalogue number: 201905, Elabsciences), adiponectin ELISA kit (catalogue number: E-BC-K013-S, Elabsciences), DPP-4 ELISA kit (catalogue number: ab133081, Abcam, Waltham, MA, USA), catalases ELISA kit (catalogue number: E-BC-K106, Elabsciences), glutathione peroxidae ELISA kit (catalogue number: E-EL-R2491, Elabsciences), superoxide dismutase ELISA kit (catalogue number: E-BC-K020, Elabsciences), malondialdehyde ELISA kit (catalogue number: E-EL-0060, Elabsciences), α-Amylase ELISA kit (catalogue number: E-EL-R2544, Elabsciences), α-glucosidase ELISA kit (catalogue number: E-EL-R1083, Elabsciences), hexokinase ELISA kit (catalogue number: E-EL-RR0502, Elabsciences), glucose 6 phosphatase ELISA kit (catalogue number: E-EL-M1362, Elabsciences), GLUT-4 ELISA kit (catalogue number: E-EL-RR0430, Elabsciences), TNF-α ELISA kit (catalogue number: E-EL-R0019, Elabsciences), and IL-6 ELISA kit (catalogue number: E-EL-R0015, Elabsciences).

### 3.2. Experimental Design

For this study, a total of thirty adult Wistar male albino rats, weighing between 150 and 200 g, were carefully selected. These rats were housed in the animal facility of the university, providing a suitable environment for their well-being. The rats were accommodated in polycarbonate cages with stainless steel covers and provided with woodchip bedding, which offered comfort and a suitable surface for movement. The study was conducted for four weeks.

The animal facility maintained optimal conditions to ensure the rats’ health and minimize any external factors that could influence the experiment’s outcomes. The temperature within the facility was kept constant at 25 ± 5 °C, creating a comfortable and stable environment. The relative humidity was maintained at 50 ± 10%, providing adequate moisture levels for the rats’ well-being. Appropriate light and dark cycles were implemented to simulate natural day and night conditions, promoting regular circadian rhythms.

To meet their nutritional needs, the rats were provided with standard pellet food and water ad libitum, meaning they had unrestricted access to food and water throughout the study. The food provided met the required nutritional standards for laboratory rats, ensuring a balanced diet. The thirty rats selected for the experiment were divided into three groups as follows:

(1) Control group: This group received distilled water, serving as the baseline reference for comparison.

(2) Group 2: This group received BPA at a dosage of 70 mg/kg/day.

(3) This group received a combination of BPA at a dosage of 70 mg/kg/day and RSV at a dosage of 100 mg/kg/day.

Preparation of stock solution of BPA: BPA is available in a small white pellet form. Its stock solution was prepared by following the method as described previously [34].

Preparation of stock solution of RSV: RSV is available in the form of a coarse powder and its solubility in water is limited but is more soluble in organic solutions. For the administration of the RSV into the experimental animals, RSV was blended in corn oil and the concentration of RSV was adjusted in the corn oil according to the body weight of the experimental animals by following the method as described previously [34] with some modifications.

Optimum selection of RSV dose: The dose of RSV (100 mg/kg/day) was selected on the basis of the beneficial effects of the RSV as described previously [35,36]. Moreover, in our study, we also observed that when we administered RSV blended in corn oil to the normal rats, we did not observe any alterations in the metabolomic profiling of the normal rats [34]. Therefore, we did not choose a separate experimental rat group to investigate the any possible toxicity and/or effects of RSV on the normal metabolic processes of healthy rats.

The administration of BPA and/or RSV to the rats continued for a period of four weeks. Throughout the study, the rats’ food and water intake were closely monitored on a daily basis. This monitoring aimed to ensure that the rats consumed the administered substances and maintained their usual dietary patterns. By tracking their food and water intake, any potential variations or effects on the rats’ overall well-being could be observed and considered during the analysis of the experiment’s results.

### 3.3. Evaluation of Anthropometric Markers

In this study, anthropometric markers were used to assess the body weight and body mass index (BMI) of the experimental rats. The body weight of each rat was measured at the beginning of the experiment (week 0) and then monitored weekly throughout the study period. This allowed us to track any changes in body weight over time and evaluate the impact of the experimental conditions on the rats’ growth and development. The formula used to calculate the BMI of rats in this study was as follows:

BMI = Body weight (g)/body length^2^ (cm^2^)

By plugging in the recorded body weight and body length values into this equation, we were able to obtain the BMI values for each rat at the start and end of the experiment. By monitoring both the body weight and BMI of the rats throughout the study, we gained important insights into the physical changes and growth patterns of the experimental animals. These anthropometric markers served as valuable indicators of the rats’ response to the experimental conditions and provided quantitative data for evaluating the effects of the study interventions on their body composition.

### 3.4. Biochemical Analysis

#### 3.4.1. Evaluation of Glycemic Control Biomarkers

In this study, we aimed to evaluate glycemic control by assessing several important biomarkers, including serum insulin level (measured in mU/L), plasma glucose level (measured in mmol/L), HbA1c (measured in mmol/mol), and HOMA-IR.

To measure rat blood glucose levels, we employed the glucose strip method. The estimation of serum insulin, HbA1c, and DPP-4 levels in rats was conducted using ELISA kits in accordance with the guidelines provided by the manufacturers. To quantify the optical density (OD) measurements, a microplate ELISA (Bio Tek instrument, Inc., Winooski, VT, USA) was employed. Additionally, to calculate the HOMA-IR, we utilized fasting values of glucose and insulin. HOMA-IR is a widely used index for assessing insulin resistance. It is calculated using the following formula:

HOMA-IR = [glucose (nmol/L) × insulin (µU/mL)/22.5]

By plugging in the fasting values of glucose (measured in nmol/L) and insulin (measured in µU/mL) into this formula, we were able to obtain a numerical value representing the degree of insulin resistance. Overall, through the combination of these measurement methods and calculations, we gained valuable insights into the glycemic control and insulin sensitivity of the study participants, providing a comprehensive understanding of their metabolic health.

#### 3.4.2. Evaluation of Lipid Profile Biomarkers

In this study, the serum levels of important lipid profile biomarkers, including free fatty acids (FFAs), high-density lipoprotein (HDL), and triglycerides (TGs), were meticulously measured to gain insights into lipid metabolism. To assess these biomarkers accurately, calorimetric assay kits specifically designed for quantifying FFAs, HDL, and TGs in serum samples were used.

To perform the analysis, we employed the Micro-lab 300 chemistry analyzer. By using the calorimetric assay kits in conjunction with the Micro-lab 300 chemistry analyzer, we were able to obtain precise measurements of FFAs, HDL, and TGs in the serum samples. These measurements provided valuable information about the lipid profile of the study participants, enabling a deeper understanding of lipid metabolism and its potential implications in various health conditions, such as cardiovascular diseases and metabolic disorders.

#### 3.4.3. Evaluation of Cholesterol Biosynthesis Biomarker

In this study, the hepatic cholesterol biosynthesis biomarker, HMG-CoA reductase, was carefully assessed from the serum to understand its activity. We employed the ELISA kit assay method to evaluate the serum levels of HMG-CoA reductase.

#### 3.4.4. Evaluation of Biomarkers of Carbohydrate Metabolism

We also evaluated the serum levels of vital biomarkers directly associated with carbohydrate metabolism. By focusing on the key enzymes involved in this metabolic pathway, namely α-amylase, α-glucosidase, hexokinase, and glucose-6-phosphatase, we sought to unravel valuable insights into the intricate workings of carbohydrate utilization within the body. To accomplish this, we employed the ELISA method to accurately quantify the serum levels of these biomarkers, which allowed us to determine the OD at a specific wavelength of 450 nm. By examining the serum levels of α-amylase, α-glucosidase, hexokinase, and glucose-6-phosphatase, we were able to gain comprehensive insights into the activity and levels of these crucial enzymes involved in carbohydrate metabolism.

#### 3.4.5. Evaluation of Inflammatory Biomarkers

In addition to the extensive assessments mentioned above, we further investigated the serum levels of crucial inflammatory biomarkers including TNF-α (Tumor Necrosis Factor-alpha), IL-6 (Interleukin-6), adiponectin, and leptin to gain a comprehensive understanding of the inflammatory status and potential metabolic implications in the study subjects. To quantify the serum levels of these inflammatory biomarkers, we employed the widely employed ELISA method. To capture the measurements, we utilized a microplate ELISA reader and recorded the OD readings at a specific wavelength of 450 nm. By measuring the levels of these inflammatory biomarkers, we aimed to gain valuable insights into the inflammatory status of the study subjects.

### 3.5. Preparation of Tissue Homogenates

At the end of the treatment period, the muscle tissues were collected for further analysis. To ensure the preservation of tissue integrity, the muscle samples were promptly isolated and placed in an icebox. This step was crucial in preventing degradation and maintaining the quality of the samples.

To prepare tissue homogenates for subsequent analysis, the muscle samples were homogenized using a tissue homogenizer. The homogenization process involved combining the muscle samples with 0.01 M phosphate buffer at a ratio of 1 g of tissue per 9 mL of buffer. The purpose of this step was to break down the muscle tissues and create a uniform suspension of cellular components in the buffer solution.

Following the homogenization step, the resulting homogenates were incubated on ice for 10 min. This incubation period was necessary to ensure proper sample stabilization and allow any enzymatic reactions to subside. Cooling the homogenates on ice helped to maintain the integrity of the cellular components and prevent further enzymatic activity that could affect the subsequent analysis. After the incubation period, the homogenates underwent centrifugation at 14,000× *g* for 10 min. This centrifugation step aimed to separate the cellular debris from the clear supernatants containing the desired components for analysis. By subjecting the homogenates to high centrifugal forces, the cellular debris settled at the bottom of the tubes, allowing for the extraction of the clear supernatants. Carefully extracting the clear supernatants, these samples were then utilized for performing ELISA tests. To ensure accurate and reliable results, the ELISA tests were performed following the instructions provided by the manufacturers of the ELISA kits.

#### 3.5.1. Evaluation of Insulin-Regulated Glucose Transporter

In this significant study, we aimed to assess the levels of GLUT-4 (Glucose Transporter 4) in skeletal muscle tissue homogenates using the ELISA method. By quantifying the levels of GLUT-4 in skeletal muscle tissue, we aimed to gain valuable insights into its role in carbohydrate metabolism.

#### 3.5.2. Evaluation of Oxidative Stress and Lipid Peroxidation

The levels of oxidative stress biomarkers were meticulously assessed in liver tissue homogenates to gain insights into the extent of oxidative stress (catalase, glutathione, and superoxide dismutase) and lipid peroxidation (MDA) using the ELISA kit assay method. By evaluating these biomarkers collectively, we aimed to obtain a comprehensive assessment of oxidative stress and lipid peroxidation levels in the liver tissue homogenates, shedding light on the potential impact of oxidative stress on cellular health and function.

### 3.6. Evaluation of the mRNA Expression of the Transcriptional Regulation of Glucose Metabolism and Insulin Secretion

To obtain the RNA from the pancreas samples, we utilized TRIzol reagent (Biobasic BS410A-MA18DR0J) following the TRIzol method. This widely used method enabled the efficient extraction of total RNA from the tissue samples, ensuring its integrity and preserving the RNA’s quality for downstream analysis. To assess the quality and integrity of the extracted RNA, we performed gel electrophoresis using a 2% agarose gel. For the reverse-transcription process, 2 μg of the extracted total RNA was converted into single-stranded complementary DNA (cDNA) using the Revert Aid cDNA synthesis kit (Thermo Scientific, USA). To quantify gene expression, real-time polymerase chain reaction (qPCR) was performed. The qPCR thermal cycling was conducted under the following conditions: an initial denaturation step at 95 °C for 5 min, followed by 40 cycles of denaturation at 95 °C for 15 s, annealing at 60 °C for 20 s, and extension at 72 °C for 20 s. These cycling conditions ensured the amplification of specific DNA sequences and enabled accurate quantification of gene expression levels. We employed a Bio-Rad real-time PCR machine to perform the real-time PCR analysis.

In Table 1, we present the sequence and size information of the expected PCR products for the reference and target genes (GCK, UCP2, IGF-1, and GLUT2). These genes were carefully selected based on their relevance to the study and their potential involvement in pancreatic function and metabolism. Assessing the expression levels of these genes provided valuable insights into their potential role in the pancreatic function and metabolic processes under investigation.

These genes are known to play significant roles in pancreatic function, glucose metabolism, and insulin regulation. To ensure the accurate normalization of mRNA and cDNA quantities, the GADPH (Glyceraldehyde-3-Phosphate Dehydrogenase) gene was chosen as the internal reference gene or housekeeping gene. The GADPH gene is commonly used as a reference gene due to its stable expression across different tissues and experimental conditions.

To calculate ΔCT, the average CT value obtained from triplicate measurements of both the target gene and the reference gene (GADPH) was utilized. The difference between the ΔCT values of the control (baseline) and the sample (experimental condition) was then calculated to determine ΔΔCT. This parameter provides an estimate of the variation in gene expression levels between the control and sample. The fold changes in mRNA expression were calculated using the 2^(−ΔΔCT) method, which allows for the quantification of relative changes in gene expression. The equation used was:

ΔΔCt = (Cttarget gene − CtGADPH) BPA − (Cttarget gene − CtGADPH) control.

### 3.7. Histopathological Evaluation

After the completion of the 4th week of the experimental period, the animals were sacrificed under anesthesia. Pancreas were isolated and preserved for biochemical and histopathological analysis. For histopathological analysis, a 10% formalin solution was prepared, and tissues were preserved in this solution. The tissues were washed with distilled water for 6 h, dehydrated using 70% and 90% alcohol for 120 min, and absolute alcohol overnight. After being treated with xylene twice for 80 min, the tissues were dipped into paraffin wax overnight. Tissue blocks were prepared by placing these tissues in paraffin wax into metal plates of cube shape. Using a microtome, tissue ribbons with a thickness of 5 µm were sectioned, and the 5th ribbon section was collected and placed in a water bath at 40 °C. These sections were placed on clean slides for hematoxylin staining. They were then treated with xylene I (5 min) and xylene II (2 min) to remove the paraffin wax. The tissues were hydrated by treating them with 95% ethanol for 2 min, 70% ethanol for 3 min, and 50% ethanol for 5 min. After washing with tap water, they were treated with a sodium bicarbonate bluing solution. These sections were counterstained with eosin dye and dehydrated by increasing concentrations of ethanol (50%, 70%, and 95%). Finally, the slides were cleaned with xylene and observed under a microscope.

### 3.8. Statistical Analysis

The biochemical parameters were assessed, and their values were determined and reported as the mean ± standard deviation (SD) and GraphPad Prism Software, version: 5 (GraphPad Software Inc., San Diego, CA, USA) was used to analyze the statistical significance of the results. A significance level of *p* < 0.05 was established to determine the statistical significance of the findings. To further examine the differences between the groups, the post hoc Bonferroni test was employed for both one-way and two-way comparison analysis.

## 4. Discussion

**Figure 11:**
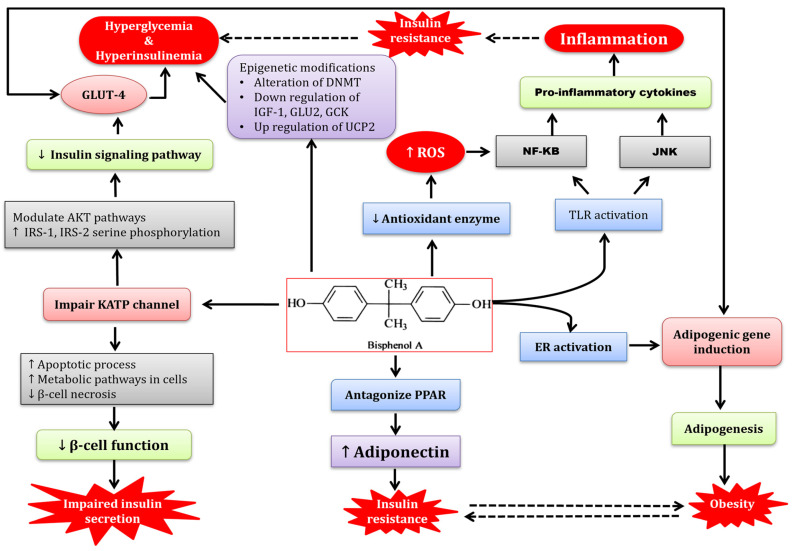
Mechanism of BPA-induced impaired metabolic pathways and its amelioration with RSV. BPA-intoxication-induced impaired carbohydrate and lipid metabolism occurs, possibly via the activation of various intracellular signal transduction pathways.

BPA is a chemical compound commonly used in the production of plastics and epoxy resins. It has been a topic of concern due to its potential adverse effects on human health. Numerous studies have investigated the role of BPA in metabolic impairment, particularly in relation to obesity and insulin resistance. Figure 11 provides a concise summary of the intricate interactions between BPA and various metabolic and signaling pathways, ultimately culminating in the development of diverse metabolic disorders. The diagram visually represents the complex relationship between BPA exposure and its impact on metabolic parameters.

Mitochondrial UCP2 is a crucial component within the β-cells of pancreatic islets. Numerous studies have established a notable correlation between the overexpression of UCP2 and hyperinsulinemia, highlighting its involvement in the development of metabolic disorders [37,38]. As a result, the regulation of UCP2 levels becomes of utmost importance, as disturbances in its expression can disrupt essential metabolic processes. Another key element in maintaining glucose homeostasis is the insulin-responsive glucose transporter known as GLUT4. Primarily expressed in skeletal muscles, GLUT4 plays a vital role in facilitating the uptake of glucose into cells. Dysregulation and impairment of GLUT4 function contribute significantly to the development of insulin resistance [39,40]. Interestingly, while GLUT2 is predominantly identified in hepatocytes, it is also present in the pancreas, where it plays a crucial role in glucose-stimulated insulin secretion. GLUT2 enables the diffusion of glucose across cell membranes, initiating intracellular glucose metabolism through the action of the enzyme GCK. Previous findings have demonstrated a strong association between the reduced expression of GLUT2 and impaired glucose-stimulated insulin secretion in diabetic rodents [41]. IGF-1, a polypeptide hormone composed of 70 amino acids, plays a significant role in glucose metabolism and homeostasis. The downregulation of IGF-1 levels has been shown to have hyperglycemic effects, further emphasizing its importance in maintaining proper glucose regulation [42].

In our study, we observed that BPA exposure significantly elevated blood glucose levels (Figure 2). This finding is further supported by the increased serum levels of HbA1c, a marker of long-term blood glucose control (Figure 3B), and HOMA-IR, an index of insulin resistance (Figure 3C). These results highlight the disruptive effects of BPA on glucose homeostasis and insulin sensitivity, contributing to the development of metabolic disorders. To further support the potential of RSV as a therapeutic intervention, we refer to a study conducted by Liu et al. [43] that investigated the impact of RSV on blood sugar levels and insulin sensitivity. Their findings demonstrated that RSV exhibited notable improvements in glucose homeostasis and insulin sensitivity. These findings align with our observations and provide additional evidence for the beneficial effects of RSV in managing metabolic dysfunctions.

Collectively, the results from our study and the referenced study emphasize the detrimental effects of BPA on metabolic parameters and highlight the potential of RSV as a therapeutic agent for mitigating the disturbances caused by BPA. By improving glucose regulation and enhancing insulin sensitivity, RSV holds promise as a beneficial intervention in managing metabolic dysfunctions induced by BPA exposure. These findings provide valuable insights into the potential application of RSV as a protective and therapeutic strategy in the context of BPA-related metabolic disorders. Lipid peroxidation is a process resulting from the oxidation of lipoproteins and lipids, which can lead to cellular apoptosis and an increase in free radicals. Serum levels of MDA are often used as an indicator of lipid peroxidation and oxidative stress [44]. In our study, we found that BPA exposure led to elevated MDA levels (Figure 6D), indicating an increase in free radicals.

To further understand the impact of BPA on the liver, we examined the levels of antioxidants. Our findings revealed a significant decrease in CAT levels (Figure 6A), GPx levels (Figure 6B), and SOD levels (Figure 6C) in the BPA-exposed group. These results indicate that BPA stimulates the production of ROS and disrupts the liver’s antioxidant defense system. Previous research has demonstrated the beneficial effects of RSV treatment in reducing serum MDA levels, increasing SOD levels, enhancing GSH and GPx levels, and reducing ROS levels in liver tissues. Consistent with these findings, our study observed similar outcomes in liver tissue homogenates, further validating the potential of RSV in counteracting BPA-induced oxidative stress. In addition to oxidative stress, we investigated the expression of key genes involved in glucose metabolism and insulin regulation. GCK, a crucial enzyme involved in glucose metabolism and insulin release, plays a significant role in maintaining glucose homeostasis. We observed that BPA suppressed the expression of GCK, while RSV treatment improved its expression (Figure 8).

IGF-1, another factor that stimulates insulin release, was also impacted by BPA exposure. We found a significant suppression of IGF-1 expression in response to BPA exposure, while RSV treatment increased its mRNA expression (Figure 8), consistent with previous studies. These findings suggest that BPA disrupts insulin regulation and glucose homeostasis, while RSV treatment shows potential in restoring their balance. UCP2, a critical player in lipid and energy metabolism, is associated with obesity and hyperinsulinemia. In our study, we observed an increase in UCP2 expression following BPA exposure (Figure 9), aligning with previous research. This upregulation of UCP2 may contribute to the disruption of mitochondrial acetyl-CoA oxidation and energy metabolism.

Furthermore, our study also observed the downregulation of GCK and GLUT2 expression in the pancreas of BPA-treated groups, which can disrupt normal glucose metabolism. However, RSV treatment showed potential in restoring their activity, as seen by the augmentation of GCK and GLUT2 expression. These findings provide insights into the impact of BPA on key genes involved in carbohydrate metabolism and insulin regulation and highlight the potential of RSV in restoring their function.

Overall, our study demonstrates the detrimental effects of BPA-induced oxidative stress on liver tissue and the potential therapeutic role of RSV in counteracting these effects by effectively neutralizing free radicals. Moreover, the dysregulation of key genes involved in carbohydrate metabolism and insulin regulation further emphasizes the impact of BPA on metabolic pathways, while highlighting the potential of RSV in restoring their normal activity. RSV, through its various mechanisms of action, exerts effects on glucose homeostasis by upregulating the expression of GLUT2 and GCK genes. This regulation is mediated through the activation of SIRT1 genes, which play a crucial role in maintaining glucose balance and positively regulating glucose-stimulated insulin secretion in pancreatic β-cells. The activation of SIRT1 by RSV can increase insulin secretion and ATP production by suppressing the transcriptional activity of the UCP2 gene. Previous studies have also suggested that the RSV-mediated downregulation of UCP2 occurs through SIRT1-dependent activation. Therefore, RSV activates SIRT1, which in turn represses UCP2 expression.

In our study, we observed that RSV treatment resulted in an increase in the expression of GLUT2 and GCK genes, which are important for glucose metabolism and insulin regulation. These findings indicate that RSV has the potential to enhance glucose uptake and utilization, leading to improved glucose homeostasis. Furthermore, RSV treatment led to a decrease in the expression of UCP2, which is associated with improved mitochondrial function and energy metabolism. These findings support the notion that RSV can modulate gene expression related to glucose metabolism and contribute to the downregulation of UCP2 expression. The regulation of these key genes by RSV provides insights into the molecular mechanisms underlying its beneficial effects on metabolic disorders induced by BPA exposure.

However, it is important to consider the limitations of our study. While our findings provide valuable insights, they should be interpreted within the context of the study’s limitations. Further research is needed to validate these results and expand our understanding of the underlying mechanisms. Future studies should involve human subjects to confirm the translatability of the findings. Additionally, elucidating the specific signaling pathways and molecular mechanisms through which RSV exerts its effects would provide a more comprehensive understanding of its therapeutic potential. Long-term studies are also necessary to assess the sustained effects of RSV treatment. Exploring additional pathways and optimizing the dosage and duration of RSV treatment are important for maximizing its therapeutic benefits.

## 5. Conclusions

The data presented in this study highlight the detrimental effects of BPA exposure on metabolic homeostasis and provide evidence for the potential of RSV as a therapeutic intervention to mitigate these disruptions. The findings demonstrate that BPA induces inflammation, impairs carbohydrate metabolism, causes histopathological changes in pancreatic and liver tissues, induces oxidative stress, and disrupts gene expression related to glucose and lipid metabolism. However, RSV treatment shows promise in counteracting these adverse effects. RSV effectively reduces inflammation by restoring the balance of inflammatory adipocytokines. It improves carbohydrate metabolism by upregulating the expression of GLUT2 and GCK genes, crucial for glucose homeostasis. RSV also ameliorates the histopathological changes in the pancreas and liver, indicating its protective effects on these tissues. Furthermore, RSV exhibits potent antioxidant properties, scavenging free radicals and restoring the activity of key antioxidant enzymes. The modulation of gene expression by RSV is another significant finding. It upregulates GLUT2 and GCK genes while downregulating UCP2, suggesting its beneficial effects on glucose and lipid metabolism. These results collectively highlight the potential of RSV as a therapeutic agent in managing BPA-induced metabolic disorders. However, it is important to acknowledge the limitations of the current study, including the need for further research involving human subjects, the elucidation of underlying mechanisms, long-term studies, the exploration of additional pathways, and the optimization of dosage and duration. Overall, the findings underscore the importance of addressing the adverse effects of BPA on metabolic pathways and provide valuable insights into the potential of RSV as a promising intervention to restore metabolic homeostasis. Further research in this field will contribute to a better understanding of the therapeutic mechanisms and optimal utilization of RSV to counteract the detrimental effects of BPA on metabolism.

## Figures and Tables

**Table 1:** List of primers employed in the qRT-PCR analysis of the targeted genes.

Target Gene	Primers	Sequence 5′ to 3′	Accession Number
GCK	Forward	TGGTTCCTGTCCACCATTAGTT	XM_006251179.4
	Reverse	CCAGGTCAGTGCCTTAGTGC	
UCP-2	Forward	GCCAACCTCATGACAGACGA	NM_019354.3
	Reverse	AGGAAGGCATGAACCCCTTG	
IGF-1	Forward	GCTCCAAAGCAGACAAAATACCC	XM_0391022225.1
	Reverse	GGTCTGGGCACAAAGATGGA	
GLUT-2	Forward	GCAGCCTTGGTTAAGAAGGTCA	XM_039101783.1
	Reverse	CTTCTGACATGTTGCGTGCG	

## Data Availability

All data are available within the manuscript.
